# Corticospinal Control of Human Locomotion as a New Determinant of Age-Related Sarcopenia: An Exploratory Study

**DOI:** 10.3390/jcm9030720

**Published:** 2020-03-06

**Authors:** Federico Gennaro, Paolo Maino, Alain Kaelin-Lang, Katrien De Bock, Eling D. de Bruin

**Affiliations:** 1 Department of Health Sciences and Technology, Institute of Human Movement Sciences and Sport, ETH Zurich, 8093 Zurich, Switzerland; katrien-debock@ethz.ch (K.D.B.); eling.debruin@hest.ethz.ch (E.D.d.B.); 2 Pain Management Center, Neurocenter of Southern Switzerland, Regional Hospital of Lugano, 6962 Lugano, Switzerland; paolo.maino@eoc.ch; 3 Neurocenter of Southern Switzerland, Regional Hospital of Lugano, 6900 Lugano, Switzerland; alain.kaelin@eoc.ch; 4 Faculty of Biomedical Sciences, Università della Svizzera Italiana, 6900 Lugano, Switzerland; 5 Medical faculty, University of Bern, 3008 Bern, Switzerland; 6 Department of Neurobiology, Division of Physiotherapy, Care Sciences and Society, Karolinska Institutet, 171 77 Stockholm, Sweden

**Keywords:** sarcopenia, dynapenia, corticomuscular coherence, corticospinal control, connectivity, EEG, EMG, gait, walking, locomotion

## Abstract

Sarcopenia is a muscle disease listed within the ICD-10 classification. Several operational definitions have been created for sarcopenia screening; however, an international consensus is lacking. The Centers for Disease Control and Prevention have recently recognized that sarcopenia detection requires improved diagnosis and screening measures. Mounting evidence hints towards changes in the corticospinal communication system where corticomuscular coherence (CMC) reflects an effective mechanism of corticospinal interaction. CMC can be assessed during locomotion by means of simultaneously measuring Electroencephalography (EEG) and Electromyography (EMG). The aim of this study was to perform sarcopenia screening in community-dwelling older adults and explore the possibility of using CMC assessed during gait to discriminate between sarcopenic and non-sarcopenic older adults. Receiver Operating Characteristic (ROC) curves showed high sensitivity, precision and accuracy of CMC assessed from EEG Cz sensor and EMG sensors located over Musculus Vastus Medialis [Cz-VM; AUC (95.0%CI): 0.98 (0.92–1.04), sensitivity: 1.00, 1-specificity: 0.89, *p* < 0.001] and with Musculus Biceps Femoris [Cz-BF; AUC (95.0%CI): 0.86 (0.68–1.03), sensitivity: 1.00, 1-specificity: 0.70, *p* < 0.001]. These muscles showed significant differences with large magnitude of effect between sarcopenic and non-sarcopenic older adults [Hedge’s g (95.0%CI): 2.2 (1.3–3.1), *p* = 0.005 and Hedge’s g (95.0%CI): 1.5 (0.7–2.2), *p* = 0.010; respectively]. The novelty of this exploratory investigation is the hint toward a novel possible determinant of age-related sarcopenia, derived from corticospinal control of locomotion and shown by the observed large differences in CMC when sarcopenic and non-sarcopenic older adults are compared. This, in turn, might represent in future a potential treatment target to counteract sarcopenia as well as a parameter to monitor the progression of the disease and/or the potential recovery following other treatment interventions.

## 1. Introduction

Sarcopenia is an age-related progressive decline in skeletal muscle mass and function [1], which was only very recently officially recognized as a muscle disorder by the World Health Organization (WHO) with a specific code (M62.84) from the 10th revision of the International Classification of Diseases (ICD-10) [2]. Since the first term of sarcopenia was coined in 1988 [3], several sarcopenia definitions have been developed, among which the most used were developed by the European Working Group on Sarcopenia in Older People (EWGSOP) [4,5], the International Working Group on Sarcopenia (IWGS) [6], the Society of Sarcopenia, Cachexia and Wasting Disorders (SCWD) [7] and the Foundation for the National Institutes of Health Biomarkers Consortium Sarcopenia Project (FNIH) [8]. However, although some authors undertook comparisons between different sarcopenia algorithms [9,10,11] a global consensus on the best definition is still lacking. Moreover, the Centers for Disease Control and Prevention (CDC, USA) has recently recognized that sarcopenia is in need of improved measures for diagnosis and screening [2].

Muscle weakness is well-known to place older adults at an increased risk of mobility limitations and mortality [12] and sarcopenia can be present in up to ~20% of community-dwelling older adults above 65 years old, and up to ~50% in those aged above 80 [2,6,13]. For several decades, it was initially believed that sarcopenia was mainly due to poor muscle mass. However, recent studies demonstrated that muscle atrophy is a relatively small contributor to the loss of muscle strength, since the latter is lost at a substantially faster rate than muscle atrophy and gaining muscle mass does not necessarily prevent the aging-related loss of muscle strength [14]. For this reason, the new term *dynapenia* (from Greek: *dyna*- = power/strength and -*penia* = loss) has been suggested to replace the traditionally used term *sarcopenia* (from Greek: *sarco*- = meat/muscle and -*penia* = loss) [1,14,15,16]. Interestingly, ongoing theoretical reasoning [17,18] and mounting evidence points to changes in central nervous system (CNS) function and/or the intrinsic force-generating properties of skeletal muscle as contributors to muscle weakness and motor dysfunction [18]. That is, a not well-functioning neuromuscular system in sarcopenia might be due to an impaired corticospinal interaction [18], which, in turn, may contribute to the muscle microenvironment leading to the loss of muscle mass, strength and functionality.

Corticomuscular coherence (CMC) is a measure of synchronization between motor cortical areas and spinal motor neurons and it reflects an effective mechanism of corticospinal interaction and central drive to skeletal muscle [19,20,21,22,23]. The loss of CMC has been defined as “the hallmark of aging” [24], and a weaker synchronization between supraspinal and subspinal structures has been observed in older compared to young adults [25,26]. Walking is a precision task involving both motor and cognitive efforts which become more demanding in older adults and, thus, impaired walking is believed to be one of the causes leading to an increased fall risk [27]. Gait is a locomotor task driven by neural inputs directed to skeletal muscles [28,29]. Interestingly, sarcopenic older adults, who often exhibit cognitive impairments [16,30,31,32,33], also show gait speed decline [34]. However, the neuronal and nervous dynamics occurring in older adults during walking are only partly investigated and understood. Investigating the corticospinal role in the control of gait can be achieved within the so-called mobile brain/body imaging (MoBI) research framework [35,36,37], which encompasses concurrent recordings of brain activity, for example by means of Electroencephalography (EEG), and other neurophysiological activity; e.g., muscle activity by means of Electromyography (EMG) or biomechanical parameters. The latter can be done by employing camera-based motion capture or through kinematic assessment using inertial measurement units. MoBI can help in finding insights about the corticospinal control available during locomotion tasks and how this relates to biomechanical, neurophysiological and/or cognitive aspects [38,39,40]. Corticospinal control of gait has been assessed in clinical settings in people suffering from spinal cord injury [41], and in neuromuscular diseases and movement disorders [42,43,44,45,46]. Moreover, corticospinal control of gait has been investigated also in healthy young and older participants [28,29,47,48,49]. When different age groups are compared it seems that CMC is differently modulated in older compared to younger adults when different gait task modalities are employed [48,50]. CMC assessed during walking may represent one approach that could fit the CDC recommendations [2] on improving the current state of the art of screening for and diagnosing of sarcopenia. Furthermore, a possible neurogenic contribution in this ICD-10 recognized muscle disorder may shed a light on a potentially disrupted pyramidal system as contributing factor related to the loss of muscle mass, strength and functionality in sarcopenia.

Measures of central drive to skeletal muscle, such as CMC, have been shown to potentially serve as putative biomarker able to distinguish several neurological and neuromuscular diseases, such as stroke [51], motor neuron disease [46] and dystonia [52]. However, to the best of our knowledge it seems that using neurophysiological measures of neuromuscular functioning to distinguish between sarcopenic and non-sarcopenic individuals is so far not available. Therefore, the aim of this exploratory observational study was to investigate the corticospinal control of gait and explore its possible relation with sarcopenia by comparing older adult individuals with against those without sarcopenia. We, furthermore, explore whether corticomuscular coherence between EEG and EMG recordings over several lower limbs muscles during gait can distinguish the presence of sarcopenia.

## 2. Experimental Section

### 2.1. Participants

A total of ~2500 community-dwelling older adults were informed about the study through a letter with a pre-paid response letter send by ATTE, an association of older adults of the Canton Ticino (ATTE, Bellinzona, Switzerland) as well as through word of mouth and with the help of other local associations and institutions that were approached in person. Of these, ~500 community-dwelling older adults informed the experimenter to be interested in participating. Community-dwelling volunteers interested to participate were included if they were ≥ 65 years old, were able to walk without any walking aid and when having a body mass index (BMI) ≤ 30 kg/m^2^. Volunteers interested to participate were excluded if they had a self-reported history or clinical signs or symptoms of severe, uncontrolled or unstable diabetic, cardio-circulatory, respiratory, liver, renal, thyroidal, neurological, neuromuscular, peripheral arterial disease(s) or autoimmune disease. Moreover, they were excluded if they had recent lower limb fracture or lower limb surgeries in the previous 10 years or pain in either left or right lower limb or any other condition that could be considered a contraindication for muscle or walking/gait testing. Additionally, interested participants were excluded if they had a current or past (within 10 years) history of malignancy (excluding non-melanoma skin cancer). A total of 203 participants were included for the present study, however, of which five participants had to be excluded from further analysis for health reasons. Therefore, a total of ~198 participants (120 females; age: 73 ± 6 years; range: 65–97 years; Table 1) were included and completed all the experimental procedures. The study protocol was approved by the cantonal ethics committee of Ticino (2018-00040 CE 3316) and an informed consent in accordance with the Declaration of Helsinki was obtained signed by the participants before starting the experimental procedures.

### 2.2. Experimental Protocol

Experimental measurements were performed in two separate sessions. After signing the informed consent, height and weight were measured, and BMI was calculated for each participant dividing the weight in kilograms by the height in meters squared. Afterwards, for the purpose of screening for the presence of sarcopenia, three measurements were performed always in the following order: muscle strength, skeletal muscle mass and physical performance. In the second session, only the older adults identified as having sarcopenia (see Section 2.3) were asked to participate, together with a sample-matched control group chosen at random from the entire sample of participants (see Section 2.3. below). In this second session, CMC has been measured between EEG and EMG recorded from several lower limb muscles during overground walking (see Section 2.5.3. below).

### 2.3. Sarcopenia Screening

#### 2.3.1. Body Composition

Body composition, and specifically, skeletal muscle mass and total body fat were assessed by bioelectrical impedance analysis (BIA; Inbody 120, InBody Co., Ltd. Seoul, Korea). Skeletal muscle mass was represented by appendicular lean muscle mass (ALM), further adjusted by height squared or by BMI depending on the operational definition of sarcopenia (see Section 2.3.4. below). Participants were asked to stand barefoot on the BIA platform while holding with both hands the handlebar connected to the device after cleaning both hand palms and foot soles provided for this purpose by the BIA manufacturer. When possible, participants were asked to remove metallic objects and participants having a pacemaker were excluded from body composition and further measurements. Although BIA is not considered the “gold standard” muscle mass measuring method, it has been accepted as an alternative method to the reference (and more valid) dual-energy X-ray absorptiometry (DXA), representing a good trade-off between method validity and clinical practice convenience [4,5,53,54].

#### 2.3.2. Muscle Strength

Muscle strength was assessed using a handheld dynamometer (Saehan Co., Yangdeok-Dong, Masan, South Korea). Participants were comfortably seated holding the handgrip with the left/right hand with wrist in neutral position, thumb facing upward and resting the arm and forearm over the ipsilateral leg. The experimenter was always the same person seated in front of the participant and supporting the weight of the dynamometer during each muscle strength trial. The participant was asked to perform an isometric maximum voluntary contraction with each side (left/right) for ~5 s. Each participant always started with the right side and continued with the left-side as soon as the first side trial was terminated. Participants rested ~120 s between right-left side pairs of trials and a total of three (pairs) of trials were performed. The highest value from all trials was used as muscle strength value. Verbal encouragement was provided during each muscle strength trial in a standardized fashion.

#### 2.3.3. Physical Performance

Physical performance was assessed by measuring gait speed over a course of ~5 m. Participants were asked to walk at their natural self-paced gait speed by wearing comfortable shoes usually worn in their daily walking activities. High-heel shoes were not allowed for the gait speed test. The operator gave verbal instruction relative to both start and end of the gait trial, which was considered completed when participants performed three times the entire gait course of ~5 m. Gait speed was measured and recorded with an inertial measurement unit (G-WALK, BTS Bioengineering, Milan, Italy) placed on the lower back at the height of the second lumbar spinal process below the imaginary line connecting the left and right most prominent bone of the posterior superior iliac spine.

#### 2.3.4. Operational Definition of Sarcopenia

Given that a “gold standard” operational definition of sarcopenia is still lacking, we decided to opt for using a multi-algorithms strategy to define and detect “sarcopenic” older adults from the participants of this study, in line with previous studies [55,56]. More specifically, we used the 1st and 2nd version of the EWGSOP, respectively EWGSOP1 [4] and EWGSOP2 [5], the IWGS [6], the SCWD [7] and the FNIH [8]. All these definitions take specific thresholds into account derivable from some parts or from all the measurements of skeletal muscle mass, muscle strength and physical performance taken from the individuals (see Table 2 for further details of thresholds used). Additionally, the EWGSOP1 was further employed by using three different thresholds of ALM (see Table 2 for further details of thresholds used). We specifically used the ALM definition from Baumgartner (EWGSOP1_BAUM_) [57], the first definition from Delmonico (EWGSOP1_DELM1_) [58] and the second definition from Delmonico (EWGSOP1_DELM2_) [59].

### 2.4. Experimental Protocol

**Figure 1:**
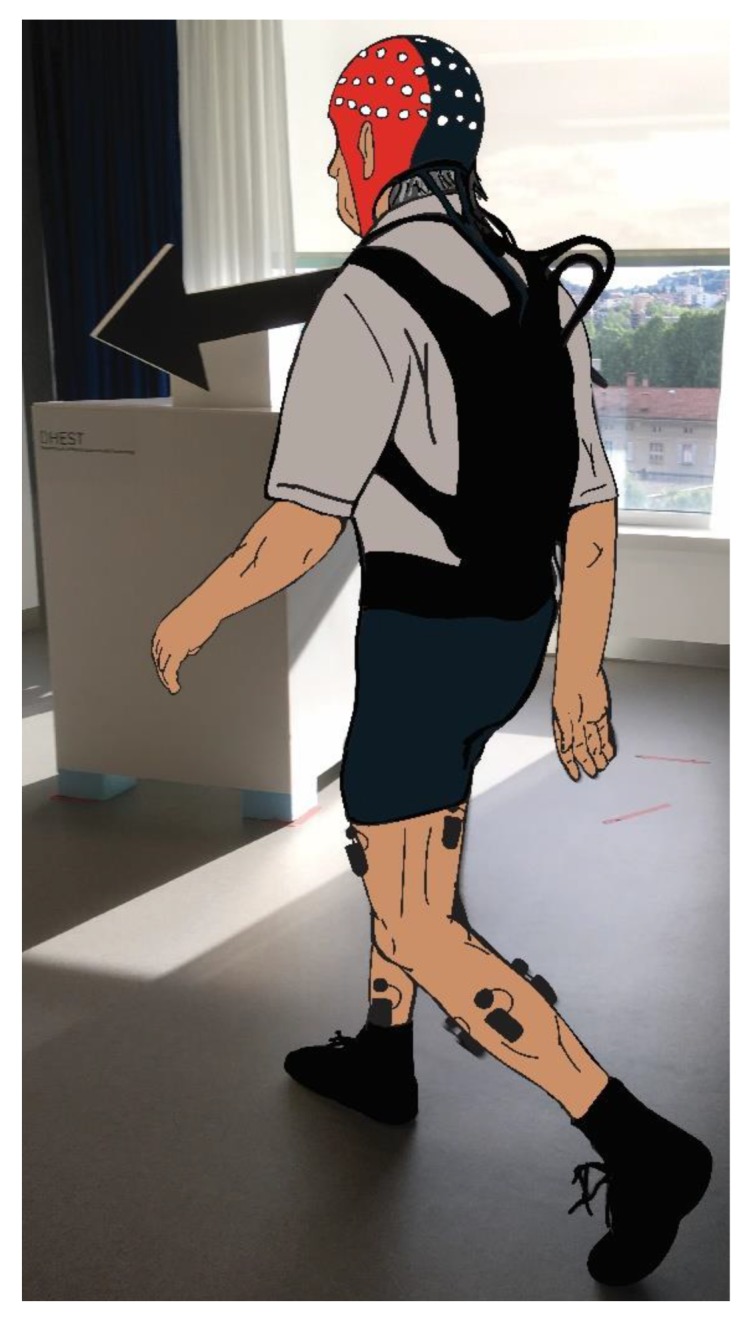
A participant is depicted while approaching to turn around one of the two parallelepiped-shaped structures with an easy-to-spot big black arrow on top showing the turning direction. The figure-8 gait path is composed by two structures as depicted and walking is performed by turning around each structure continuously. The participant walked in the figure-8 gait course, while wearing an EEG cap and EMG sensors over eight muscles of both left and right leg. Moreover, two footswitches were placed under the sole of the foot before wearing socks and shoes. The backpack served to store the amplifier of the EEG cap and additional elements (i.e., cables), however EEG signals were monitored real time remotely.

Electrophysiologic measurements were carried out only in older adults diagnosed to be sarcopenic by the operational definitions of sarcopenia and a sample-matched randomly selected control group of older adults from the participants of this study. From the seventeen sarcopenic older adults found after screening, eleven participated at the second session of the electrophysiologic measurements. From the remaining six sarcopenic older adults two participants were not able to participate for health reasons and four participants for personal reasons. Therefore, the electrophysiologic assessments were performed in eleven sarcopenic older adults and eleven healthy older adults (SARCO: *n* = 11 and CTRL: *n* = 11, respectively; see Table 3 for further details). Cortical and muscular activity was recorded during overground walking in a figure-8 gait course. The figure-8 gait course was structured by two custom-built parallelepiped-shaped structures with an in-between distance of ~5 m. Participants were asked to walk continuously without stopping by turning around each of these two structures. On top of each structure, a big easy-to-spot arrow was placed to indicate the direction and side of turning (depicted in Figure 1).

Participants started the gait trial from one of these two structures, which was always kept the same, and whenever they were comfortable and ready to start. Start was always after a verbal “start” call and subjects were expected to walk continuous until a subsequent verbal “stop” call was verbally expressed by the experimenter. Participants were asked to walk at a self-selected preferred walking speed. The gait trial was considered completed when the participant performed a total of 15 figure-8 loops. A total of three trials were performed with an in-between rest of ~5 min. Counting of figure-8 gait loops was performed by the experimenter and not by the participant in order to avoid any possible dual-task cognitive additional load. A tape was applied on the ground at ~1 m distance from the structure to manually trigger beginning and ending of straight walking parts of the gait path by manually pressing computer keyboard specific keys. Participants were instructed to walk naturally as soon as possible to maintain ecological validity of the experimental protocol, but, at the same time, they were asked to maintain their gaze straight towards the arrow placed on top of each structure in front of them as much as possible during the straight part of the walking trial. When walking the curved part of the figure-8 path no instruction relative to the gaze was provided. Before executing the gait trial, participants were asked to perform a familiarization walking trial of ~5 min as warm up, followed by a ~2 min standing EEG recordings which preceded the beginning of the first gait trial and served for further analysis and data preprocessing steps.

### 2.5. Electrophysiology

#### 2.5.1. Data Acquisition

Surface EEG activity was recorded at a sampling frequency of 1000 Hz by a high-density 64-channel EEG system (eego sport, ANT Neuro, Enschede, The Netherlands). Three EEG cap sizes were employed in order to accommodate different head circumferences (waveguard, ANT Neuro, Enschede, The Netherlands) and EEG electrodes were placed according the 10-10 international system [60]. EEG reference and ground were placed over the left and right mastoid, respectively. An electrodes impedance ≤ 5 kΩ was required before EEG recordings. Surface EMG activity was recorded at a sampling frequency of 1000 Hz (FREEEMG 1000, BTS Bioengineering, Milan, Italy) by means of pairs of bipolar Ag-AgCl electrodes (H124SG Covidien, Minneapolis, MN, USA) placed with an inter-electrodes distance of ~2 cm accordingly to SENIAM guidelines [61] over eight muscles of interest in both left and right leg: Vastus Lateralis (VL), Vastus Medialis (VM), Rectus Femoris (RF), Biceps Femoris (BF), Tibialis Anterior (TA), Soleus (SOL), Gastrocnemius Medialis (GM) and Gastrocnemius Lateralis (GL). The skin was properly cleaned and, when necessary, shaved before placing the EMG sensors. The same inertial measurement unit (G-WALK, BTS Bioengineering, Milan, Italy) used for gait speed analysis during the screening of sarcopenia in the first testing session was utilized also during this measurement but not further analyzed for the present study. Heel Strike’s onsets were detected by placing two footswitches approximately on the midpoint of the calcaneus in each foot. The two sensors were stacked on top of each other in order to provide a redundant backup of the heel strike onsets recordings, in case one of the two footswitch had technical problems. EEG and EMG recordings were synchronized by sending an analog square wave pulse to both EEG and EMG system from a custom-made device equipped with Transistor-Transistor-Logic (TTL) ports in order to align both time series in the subsequent data analysis.

#### 2.5.2. Data Pre-Processing

**Figure 2:**
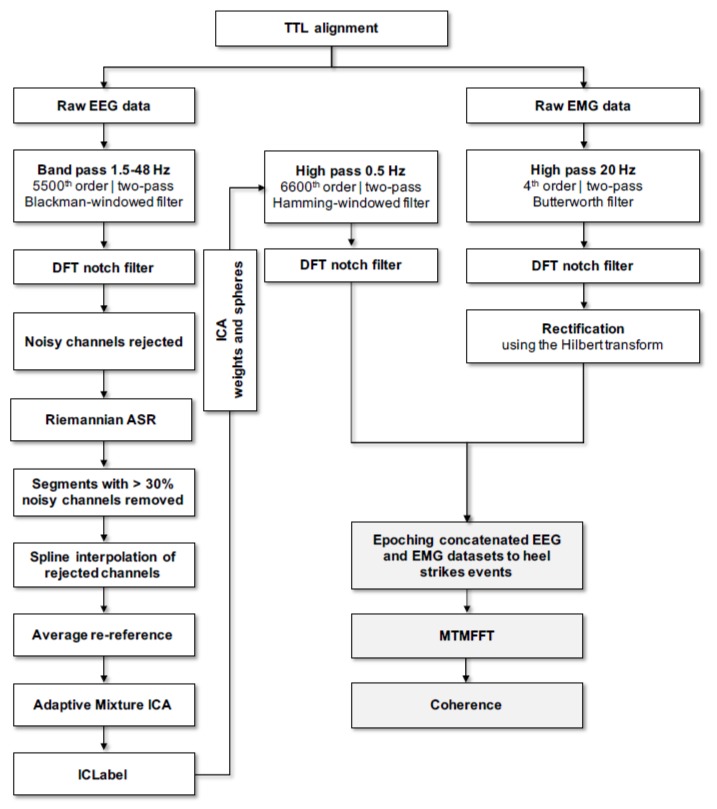
Overview of the pipeline adopted to perform data pre-processing and spectral analysis; Data pre-processing steps are depicted by the boxes, whereas spectral analysis steps are depicted by greyish boxes. TTL: Transistor-Transistor Logic; EEG: Electroencephalography; EMG: E lectromyography; DFT: Discrete Fourier Transform; ASR: Artifact Subspace Reconstruction; ICA: Independent component analysis; MTMFFT: multitaper frequency transformation.

All signal processing was performed using custom-made scripts and Fieldtrip, an open-source toolbox for electrophysiological data analysis [62] for Matlab (Mathworks Inc., Natick, MA, USA). An overview of the adopted pipeline for data pre-processing, including spectral analysis (see Section 2.5.3. below) can be retrieved in Figure 2. After alignment of the EEG and EMG data according to the TTL pulse, EMG data was high pass filtered (two pass Butterworth filter, 4th order, 20 Hz cutoff) and powerline noise, as well as its harmonics were filtered out using a notch filter based on Discrete Fourier Transformation (DFT). Filtered EMG data was then full wave rectified using the Hilbert transform as a widely used preprocessing step before undertaking further coherence analysis [63]. Only straight parts of the figure-8 gait path were hence considered for analysis of the aligned EEG/EMG data. After removing mastoid electrodes from further analysis (M1 and M2), EEG data was then bandpass filtered (two pass blackman-windowed sync filter, 5500 order, 1.5–48 Hz cutoff) and concurrently demeaned as well as detrended. Powerline noise and harmonics were filtered out as described above. Noisy channels were detected and removed if they were flat for > 5 s or the correlation between neighboring channels was < 0.6. On average, ~1 channel was removed. Artifactual activity (e.g., movement artifacts) was attenuated with the following strategy. Firstly, a non-stationary method was employed to clean occasionally large amplitude noise and increase the stationarity of EEG data in preparation of the next Independent Component Analysis (ICA) cleaning step. For this purpose, a sliding window adaptive approach based on Principal Component Analysis (PCA) decomposition was used by means of the Riemannian modified version of the Artifact Subspace Reconstruction (rASR) method [64]. The entire data was used as calibration data and a lax threshold was chosen as parameter (30 standard deviations), as previously recommended, to be large enough to reduce artifactual activity from EEG data while preserving cerebral activity [29,65]. The combined used of ICA and Artifact Subspace Reconstruction has been suggested to represent an effective strategy to remove artifactual signals from EEG data [66] and it has been largely used in studies involving cleaning of EEG data acquired during human locomotion tasks such as gait [29,37,67,68,69,70]. Portions of data not completely repaired by rASR were removed if more than 30% of channels were noisy in that data segment. Previously rejected noisy channels were then interpolated using spline interpolation and afterwards EEG data was re-referenced to an average reference and then EEG signals were decomposed into temporally maximally independent components (ICs) by applying on the remaining rank of the data Adaptive Mixture ICA (AMICA) with enabled online artifacts rejection using a threshold of five standard deviations in five iterations intervals starting after the first five iterations and the whole procedure repeated five times. AMICA algorithm was chosen given that it has been shown to outperform other ICA algorithms [71]. After AMICA, a machine learning based approach was used to identify cerebral ICs by employing the ICLabel classifier [72]. On average, ~6 cerebral ICs were identified by ICLabel which was in line with the suggested ~5–15 range of brain ICs that can be detected reliably [73]. The respective ICA weights and spheres matrices of the retained cerebral ICs were conveyed to an EEG dataset identical but processed using a more conventional filtering approach (high pass filter: two pass hamming-windowed sync filter, 6600 order, cutoff 0.5 Hz; powerline noise filtered as in the EMG analysis described above). In the present study, we have chosen to focus on the EEG Cz electrode for further spectral analysis of CMC. This vertex located sensor is widely employed to assess CMC during gait, as well as during isometric contraction tasks using lower limbs’ muscle, such as ankle dorsiflexors [28,47,48,50,74,75,76,77]

#### 2.5.3. Spectral Analysis

The cleaned preprocessed EEG and EMG data were then segmented according to the swing phase from 650-ms to 50-ms before heel strike onsets in analogy to previous studies [28,47,48,76,77], avoiding inclusion of any potentially remaining artifact due to the collision of the foot with the ground. On average, ~212 gait segments, considering the sum of any left and right heel strikes, were used for coherence estimation. Spectral analysis was performed as previously described [76]. Briefly, data segments were zero-padded up to 2 s and tapered with a variable set of discrete prolate spheroidal (Slepian) sequences by applying a multi taper frequency transform yielding to a broad 1–60 Hz frequency band power- and cross-spectra with a frequency resolution set to 1 Hz. The frequencies of interest (FOI) for this analysis focused on the entire beta frequency band (i.e., 13–35 Hz) gathered with the lower bound of the gamma frequency band (i.e., 36–48 Hz), since it appears that, at individual level, the maximum amount of coherence (e.g., peak) related to the stance phase of gait that can be present in a beta-to-lower gamma FOI (i.e., ~13–50 Hz) [28,48,78]. Therefore, we adopted ten tapers resulting in a spectral smoothing of ±9 Hz. With this strategy, we assured to encompass our beta-to-lower gamma FOI using a total bandwidth of ~18 Hz and, therefore, including the entire beta frequency band, where the entire bandwidth is usually found to be ~10 Hz, but also, partially, the gamma frequency band, where the entire bandwidth is reported to be ~25 Hz [23]. Furthermore, it has been shown that the central drive to muscles during gait is largely and broadly present in this FOI [28,41,47,48,77,79,80,81]. Coherence estimates were considered significant if they exceeded a confidence limit (CL) with a probability of 95% (α = 0.05), related to the number of segments used for the coherence calculation (i.e., heel strikes, which consisted in a variable quantity for each participant, multiplied by the number of tapers used in the multi-tapered spectral analysis). CMC was estimated for the left and right side separately, to take into account the unequal number of segments between sides and per participant. However, for further statistics analysis, the maximum CMC estimate between left and right side was used, and, in case only one side was used for spectral analysis (i.e., because of technical problems in a specific footswitch and side), then only one side was considered for further analysis.

### 2.6. Statistics

In order to test the ability of CMC in distinguishing between sarcopenic and not sarcopenic older adults, Receiver Operating Characteristics (ROC) and relative accuracy (i.e., Area Under the Curve; AUC) statistics was estimated using easyROC [82]. This statistic procedures allowed to determine precision (i.e., 1-specificity), sensitivity and AUC with the following equations:

(1) $$1-\mathrm{Specificity}=\frac{T_{p}}{T_{p}+{\;F}_{p}}$$

(2) $$\mathrm{Sensitivity}=\frac{T_{p}}{T_{p}+{\;F}_{n}}$$

(3) $$\mathrm{AUC}=\frac{T_{p}+{\;T}_{n}}{T_{p}+{\;T}_{n}+F_{n}+{\;F}_{p}}$$

where T_p_ denotes true positives (i.e., sarcopenic older adults in this study), F_p_ denotes false negative and T_n_ denotes true negatives (i.e., healthy control older adults in this study). Standard Error (SE) and Confidence Intervals of the AUC was estimated using the nonparametric approach of DeLong (1998) and significance level was set to α = 0.05. Additionally, the Yoden method has been employed in order to estimate optimal cut-off points and criterion from ROC curves. Furthermore, CMC estimates were compared between groups as well as relative effect sizes using DABEST, a data analysis strategy, which uses estimation statistics [83]. Estimation statistics is considered a superior statistic compared to dichotomous significance testing, focusing on effect sizes and relative precision [83]. *P*-values are reported respective the observed effect size and confidence intervals (CI). Estimation of the 95% CI mean difference was calculated by performing 5000 bootstrapping resamples. For the between groups comparisons, a non-parametric Mann-Whitney test was used within the Data Analysis with Bootstrapped ESTimation (DABEST) framework. Magnitude of the effect was calculated as Hedge’s g, which is similar to Cohen’s d, but corrected for small-sample bias. As in Cohen’s d, an effect size ≥ 0.2 was considered small, an effect size ≥ 0.5 medium and an effect size ≥ 0.8 was considered large.

## 3. Results

**Figure 3:**
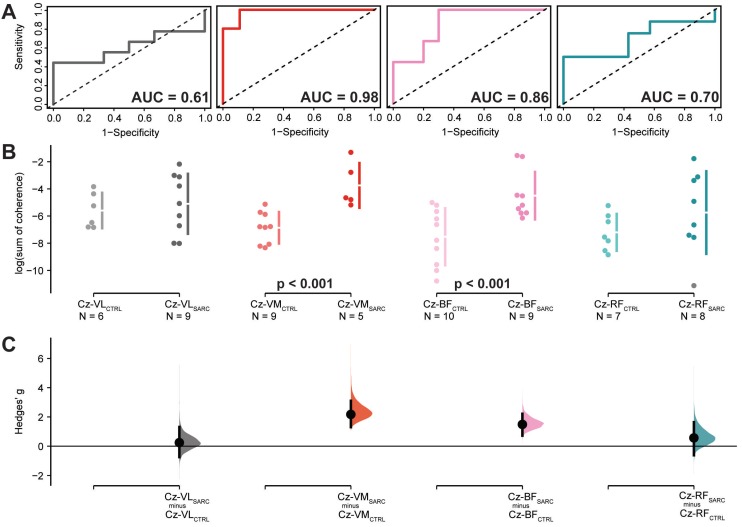
Results relative to the log-transformed coherence area (sum of coherence above significant confidence limits) between the EEG Cz sensor and the muscles located in the upper part of the lower limb: Vastus Lateralis (Cz-VL), Vastus Medialis (Cz-VM), Biceps Femoris (Cz-BF) and Rectus Femoris (Cz-RF). (**A**) shows the ROC curve with the respective accuracy (i.e., AUC estimate) depicted within the plot, precision (i.e., 1-specificity) shown on the *x*-axis while sensitivity presented in the *y*-axis. The dotted diagonal line depicts the 50% chance of differentiating between sarcopenic and non-sarcopenic older adults. (**B**) Cumming estimation plots showing mean differences of the log-transformed coherence area plotted in the upper axes separately for both sarcopenic (SARC, darkish color) and healthy control older adults (CTRL, light color). Each mean difference is represented by dots and plotted on the (**C**) lower axes as a bootstrap sampling distribution, while the ends of the vertical error bars denote the 95% confidence intervals.

**Figure 4:**
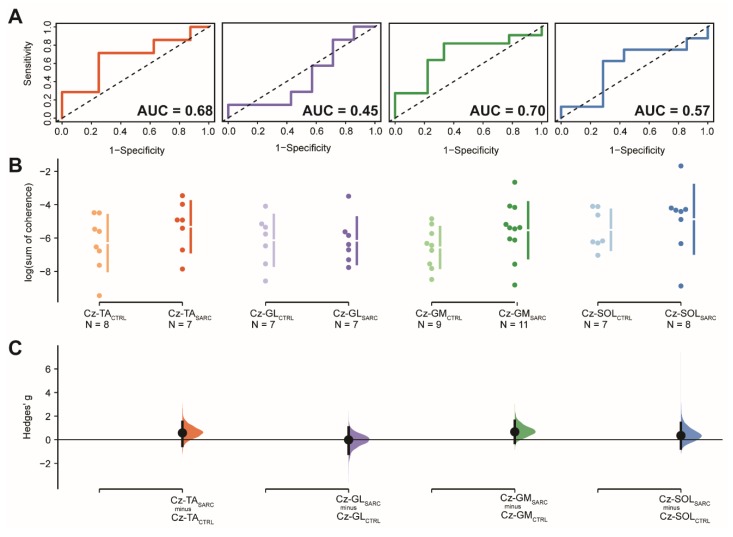
Results relative to the log-transformed coherence area (sum of coherence above significant confidence limits) between the EEG Cz sensor and the muscles located in the lower part of the lower limb: Tibialis Anterior (Cz-TA), Gastrocnemius Lateralis (Cz-GL), Gastrocnemius Medialis (Cz-GM) and Soleus (Cz-SOL). (**A**) shows the ROC curve with the respective accuracy (i.e., AUC estimate) depicted within the plot, precision (i.e., 1-specificity) shown on the *x*-axis while sensitivity presented in the *y*-axis. The dotted diagonal line depicts the 50% chance of differentitating between sarcopenic and non-sarcopenic older adults. (**B**) Cumming estimation plots showing mean differences of the log-transformed coherence area plotted in the upper axes separately for both sarcopenic (SARC, darkish color) and healthy control older adults (CTRL, lightish color). Each mean difference is represented by dots and plotted on the (**C**) lower axes as a bootstrap sampling distribution, while the ends of the vertical error bars denote the 95% confidence intervals.

ROC curve analysis of the logarithmically transformed sum of coherence above significant confidence limits, between EEG Cz sensors and eight lower limbs muscle, yielded to significant accuracy above 85% of chance in differentiating between sarcopenic and non-sarcopenic older adults, with high sensitivity and precision, when EEG Cz sensor is coupled with Vastus Medialis [Cz-VM; AUC (95.0%CI): 0.98 (0.92–1.04), sensitivity: 1.00, 1-specificity: 0.89, *p* < 0.001] or with Biceps Femoris [Cz-BF; AUC (95.0%CI): 0.86 (0.68–1.03), sensitivity: 1.00, 1-specificity: 0.70, *p* < 0.001]. For these two CMC estimates, Yoden methods suggested optimal cut-off point of the log-transformed sum of coherence for differentiating sarcopenic from non-sarcopenic older adults [−5.2 and −6.2, respectively] with an estimated cut-off optimal criterion of 0.89 and 0.70, respectively. Moreover, Cz-VM and Cz-BF showed to be significantly different between sarcopenic and not sarcopenic older adults with large effect sizes in the DABEST analysis [Hedge’s g (95.0%CI): 2.2 (1.3–3.1), *p* = 0.005 and Hedge’s g (95.0%CI): 1.5 (0.7–2.2), *p* = 0.010; respectively]. In all the other EEG Cz sensor–EMG electrode pair, ROC curves analysis did not show accuracy chance above 50% and significant or DABEST large effects sizes with significant results. ROC curve and DABEST analysis are depicted for the group of muscles located in the upper part of the lower limbs and for the group of muscles located in the lower part of the leg in Figure 3 and Figure 4, respectively. ROC curve and DABEST analysis are extensively presented for all EEG Cz sensor-muscle pairs in Table 4.

## 4. Discussion

Corticomuscular coherence represents an effective mechanism of corticospinal interaction and central drive to skeletal muscle [19,20,21,22,23] and it can be assessed during locomotor tasks such as walking [28,29,35], where it has been observed to differently modulate in older compared to younger adults [24,25,26,48,50]. Measures of central drive to skeletal muscle (e.g., corticomuscular coherence) showed to represent putative biomarkers able to distinguish several neurological and neuromuscular diseases, such as stroke [51], motor neuron disease [46], and dystonia [52]. The quality of walking has been recognized as an important biomarker of mortality and fall risk in aging [84]. The aim of this study was to perform screening of sarcopenia in community-dwelling older adults and then comparing sarcopenic with non-sarcopenic older adults using corticospinal control of locomotion by means of corticomuscular coherence between motor scalp electrocortical activity and skeletal muscles electrical activity of the lower limbs recorded during gait. The results show, to the best of our knowledge for the first time, that corticomuscular coherence between EEG Cz sensor and EMG electrodes located either on the Vastus Medialis or Biceps Femoris muscles may serve to differentiate the presence of sarcopenia with high accuracy, precision and sensitivity. The ROC results of our explorative study are supported by the observed additional results showing that CMC relative to these muscles are significantly different coupled with a large magnitude of effect when comparing sarcopenic and healthy control older adults, as shown by the Data Analysis with Bootstrapped ESTimation (DABEST) analysis. CMC is a linear measure indicating higher synchronization between motor cortical areas and skeletal muscles as it increases. This has been shown for example by observing motor learning induced increase of CMC estimates in healthy adults [85,86], but also in central drive to ankle dorsiflexors that is enhanced in older adults following a training period with exergames [80]. Both these findings are hinting towards an amelioration of the corticospinal interaction. However, one may think that an increasing value of CMC would indicate a better functioning or communicating neuromuscular system while a reduced CMC value would be pointing toward the opposite. In the available literature using similar assessment approaches this was, however, not always the case. CMC may lead to reduced CMC values in diseased populations; e.g., in stroke patients [51,87,88,89,90,91] and in Parkinson’s Disease patients [92]. Interestingly, and seemingly in contrast to some of the findings reported in literature, in our population, affected by sarcopenia, we found CMC values to be significantly larger in comparison to matched individuals without sarcopenia. This relative increased value was, furthermore, able to distinguish sarcopenic from non-sarcopenic older adults when determined from the vastus medialis and biceps femoris muscles. A possible explanation would be that sarcopenic older adults supposedly exhibit an impaired pyramidal system and a worsening of their brain-muscle communication. In this context an increase of CMC in sarcopenic older adults would be a reaction of the neuromuscular system on the sarcopenia muscle condition, that is trying to counteract the muscle weakness by increasing the synchronization efforts between motor cortical areas and skeletal muscles. This would be in accordance with findings reported for stroke patients [51,87,88,89,90,91]. Clearly this reasoning represents all but a hypothesis that should be further investigated in longitudinal studies. Some researchers hypothesize that the observed change in motor output may be due to a decline in dopaminergic output from the basal ganglia which, in turn, leads to a need to invest more cognitive resources into movements [93,94]. Other researchers hint towards the possibility of neuromuscular junctions’ dysfunction as a possible root cause with neurological origin for sarcopenia [95]. Moreover, although it has been shown that gait is driven by efferent input from motor cortical areas [28,29], disentangling any potential and additional modulations of efferent and afferent contributions to brain-muscle connectivity [35,96] might be important in order to gain a more complete picture of the neurophysiological integrity underlying sarcopenia. For instance, future investigations might consider whether a reduced afferent contribution from a progressively wasted muscle to sensorimotor areas (i.e., as in sarcopenia) might be responsible for the increased CMC observed in this study, for example as a potential compensatory mechanism. Muscle weakness is known to place elders at an increased risk of mobility limitations and mortality [12] with an incidence of 10–25% among older adults above 65 years old [6,13] and up to 50% in those aged above 80 [13]. Similar percentages account for the presence of knee osteoarthritis (KOA) [97,98,99,100,101]. Thus, KOA are usually older adults resembling muscle disuse and weakness that closely resemble the sarcopenia condition. Interestingly, it has been found that Vastus Medialis (VM) functionality (e.g., motor unit recruitment) differently modulates in KOA patients, potentially due to a compensatory mechanism in response to KOA [102]. That is, the VM structural changes observed in KOA patients are believed to be linked to neurogenic muscle atrophy [103].

Our explorative findings get support from a clinical perspective when we consider that aging associated muscle weakness underlies diverse mechanisms and cannot solely be explained by muscle atrophy [104]. The descending drive from the motor cortex declines with age, together with the overall ability to maximally activate a muscle with the nervous system, and this in sum, contributes to the decreases in voluntary contraction of muscles [18]. Voluntary activation, defined as “the level of voluntary drive during an effort” [105,106] is especially diminished in older-old individuals [107,108,109] and up to one third of losses in force production capacity may be explained by voluntary activation [110]. A recent study on cancer cachexia, which clinically presents with muscle atrophy and associated motor deficits, found that impaired neural respiratory drive was a significant contributor to respiratory muscles insufficiency [111]. Based on research findings and theoretical reasoning this has led to questioning of the conceptualizing of sarcopenia as primarily being a condition tied to the muscular system. An emerging view is that sarcopenia should be rather seen as tied to neurological factors [112]. The focus on the disease as being tied to the muscular system may also explain contradictory findings of non-pharmacological interventions used so far to prevent or control sarcopenia. Exercise programs show to be highly variable regarding type and mode of exercise offered [113] and result in low quality of evidence when summarized in a systematic review and meta-analysis [114]. Strength training for example, an exercise mode often used to treat sarcopenia, may not always be effective because it is not able to overcome the age-related blunted efferent neural drive plasticity [115]. This is independent from gains in force-generating capacity of the muscles seen when young and older master athletes are compared. Both groups may exhibit superior maximum strength and rapid muscle force production, however, the magnitude of the efferent neural drive in active older adults is substantial lower compared to that of younger individuals [109,116]. Furthermore, using a composite sarcopenia phenotype applying two different widely accepted definitions for sarcopenia does not associate with independent aging [117]. This implies that training programs should be developed that explicitly target neural structures such as training programs that use motor-cognitive approaches through step-training exergames [80,118,119,120] that also effect on leg muscles strength [121,122].

However, our findings should be considered with caution and several limitations of our study should be mentioned. Clearly the sample size is a limitation of the present study, although the large effect size found in CMC of VA and BF softens this argument. In a very recent study CMC assessed during gait revealed poor reliability within a test-retest framework [76]. However, only Tibialis Anterior (TA) has been considered in this previous work and it remains, therefore, unclear whether other muscles show different reliability patterns. Thus, further research is warranted based on these first findings from our exploratory trial. We suggest that such future research should also focus on test-retest reliability of CMC measures taken during locomotor tasks such as during walking. Another limitation may be the fact that it is not possible to guarantee that EEG data has been completely denoised from movement-related artifacts. However, it should be taken in consideration that completely denoising EEG data is almost impossible and that, for that reason, we adopted extensive care to remove artifacts from the EEG data with several precautions, such as not including data segments subsequent of heel strikes, which might have excessively contaminated EEG data by artifacts due to the impact of the foot with the ground. We also applied ASR and multi-modal AMICA algorithms as well as machine learning techniques for detecting artifactual/cerebral ICs components (i.e., ICLabel), and, in addition, adopted the strategy of transferring ICA weights and spheres to a more conventional non-ASR cleaned dataset. Both data segments (i.e., heel strikes multiplied by number of tapers used in spectral analysis) inequality between participants but also within participants (between left and right side) as well as inter-participants differences in the number of cerebral components detected by ICLabel can both represent a further limitation of the present study. However the average number of brain components detected by ICLabel were in line with the suggested range of brain components that can be reliably detected from Indipendent Component Analysis (ICA) of EEG data [73] and we estimated significant CMC at individual level using confidence limits based on the number of segments respective to each participant and specific to each side. The latter was a precaution in the coherence estimation due to the unequal number of segments between subjects but also within subjects but between left and right sides. A further limitation of this study is not having considered potential different results relative to the well-known different possible causes for Sarcopenia, and in particular primary (i.e., due to the aging process) and secondary Sarcopenia (i.e., due to muscle disuse, malnutrition or inflammation) [123]. However, this represents a further aspect for future studies wanting to explore additional features of corticospinal control of gait in relation to sarcopenia.

## 5. Conclusions

This preliminary exploratory investigation showed that corticospinal control of locomotion can represent a novel determinant in sarcopenia diagnosis, where corticomuscular coherence between EEG Cz sensor and Vastus Medialis or Biceps Femoris EMG sensors is able to distinguish between sarcopenic and non-sarcopenic older adults with high accuracy, precision and sensitivity. Moreover, our findings shed light on possible implications for future interventions either with pre-habilitation or re-habilitation purposes for the prevention and/or treatment of sarcopenia, which might employ EEG-EMG coherence, and in specific using Vastus Medialis or Biceps Femoris muscles which are shown to be significantly different with large magnitude of effect in sarcopenia, as an important parameter of the progression of the disease and/or of the potential recovery from this age-related muscle disorder.

## Figures and Tables

**Table 1:** Descriptive statistics of the study population represented as mean (± standard deviation).

	Total (*n* = 198)	Women (*n* = 120)	Men (*n* = 78)
Age (years)	73 (6)	73 (6)	74 (6)
Height (m)	1.65 (0.09)	1.61 (0.06)	1.73 (0.06)
Weight (kg)	67 (11)	62 (8)	75 (9)
BMI (kg/m^2^)	24.5 (2.8)	24.1 (2.9)	25.2 (2.6)
Total Body fat (kg)	19 (6)	20 (6)	18 (5)
ALM (kg)	19 (4)	16 (2)	24 (3)
Muscle strength (kg)	32 (10)	26 (5)	42 (7)
Gait speed (m·s^−1^)	1.08 (0.21)	1.05 (0.21)	1.14 (0.20)

ALM = Appendicular Lean Muscle Mass; BMI = Body Mass Index.

**Table 2:** EWGSOP = European Working Group on Sarcopenia in Older People; IWGS = International Working Group on Sarcopenia; SCWD = Society of Sarcopenia, Cachexia and Wasting Disorders; FNIH = Foundation for the National Institutes of Health Biomarkers Consortium Sarcopenia Project; ALM = Appendicular lean muscle mass calculated by summing lean muscle mass of upper and lower limbs; ALM/BMI = ALM adjusted by BMI; ALM/height^2^ = ALM adjusted by height squared; * ALM_PLM_ = gender-specific predicted linear model of ALM following ALM linear regression by height and total body fat mass. **♀**: ${\mathrm{ALM}}_{\mathrm{PLM}}=-13.21+14.76\times \;\mathrm{height}\;+0.23\times \;\mathrm{total}\;\mathrm{fat}\;\mathrm{mass}\;$ | **♂**: ${\mathrm{ALM}}_{\mathrm{PLM}}=-22.59+24.21\times \;\mathrm{height}\;+0.21\times \;\mathrm{total}\;\mathrm{fat}\;\mathrm{mass}.\;$

Operational Definition	Skeletal Muscle Mass ①	Muscle Strength ②	Physical Performance ③	Definition Criteria	Prevalence (%)
	Low ALM Cut-Off Points	Low Handgrip (kg)	Low Gait Speed (m/s)		
FNIH	ALM/BMI: ♀ ≤ 0.512 | ♂ ≤ 0.789	♀ < 16 | ♂ < 26	—	① + ②	—
EWGSOP1_BAUM_	ALM/height^2^: ♀ ≤ 5.45 kg/m^2^ | ♂ ≤ 7.26 kg/m^2^	♀ < 20 | ♂ < 30	<0.8	① + ② + ③	4 (~2)
EWGSOP1_DELM1_	ALM/height^2^: ♀ ≤ 5.67 kg/m^2^ | ♂ ≤ 7.25 kg/m^2^	♀ < 20 | ♂ < 30	<0.8	① + ② + ③	6 (~3)
EWGSOP1_DELM2_	ALM − ALM_PLM_ < 20th percentile of the gender-specific * distribution of residuals	♀ < 20 | ♂ < 30	<0.8	① + ② + ③	8 (~4)
EWGSOP2	ALM/height^2^: ♀ ≤ 6.00 kg/m^2^ | ♂ ≤ 7.00 kg/m^2^	♀ < 16 | ♂ < 27	≤0.8	① + ② + ③	2 (~1)
IWGS	ALM/height^2^: ♀ ≤ 5.67 kg/m^2^ | ♂ ≤ 7.23 kg/m^2^	—	<1.0	① + ③	8 (~4)
SCWD	ALM/height^2^: ♀ ≤ 5.18 kg/m^2^ | ♂ ≤ 6.81 kg/m^2^	—	<1.0	① + ③	3 (~2)
FNIH + EWGSOP1_BAUMGARTNER_ + EWGSOP1_DELMONICO1_ + EWGSOP1_DELMONICO2_ + EWGSOP2 + IWGS + SCWD					17 (~9)

**Table 3:** Descriptive statistics of the study population represented as mean (± standard deviation).

	Sarcopenic (*n* = 11)			Non-Sarcopenic (*n* = 11)		
	Total (*n* = 11)	Women (*n* = 9)	Men (*n* = 2)	Total (*n* = 11)	Women (*n* = 6)	Men (*n* = 5)
Age (years)	75 (7)	73 (6)	85 (1)	72 (4)	74 (4)	71 (4)
Height (m)	1.57 (0.09)	1.54 (0.07)	1.69 (0.04)	1.69 (0.07)	1.64 (0.07)	1.74 (0.03)
Weight (kg)	57 (9)	55 (7)	68 (12)	69 (11)	64 (9)	75 (12)
BMI (kg/m2)	23.2 (3.1)	23 (3.2)	23.9 (3.3)	24.2 (2.8)	23.7 (2.4)	24.8 (3.3)
Total Body fat (kg)	18 (5)	18 (5)	20 (9)	18 (6)	19 (5)	17 (7)
ALM (kg)	15 (3)	14 (2)	19 (2)	21 (4)	18 (3)	24 (2)
Muscle strength (kg)	23 (5)	22 (4)	31 (4)	36 (11)	28 (4)	46 (6)
Gait speed (m·s^−1^)	0.82 (0.10)	0.79 (0.09)	0.94 (0.04)	1.07 (0.08)	1.04 (0.07)	1.10 (0.08)

ALM = Appendicular Lean Muscle Mass; BMI = Body Mass Index.

**Table 4:** Descriptive Statistics of the logarithmically transformed sum of coherence above significant confidence limits reported as mean (standard deviation) and results of the Receiver Operating Characteristic (ROC) curve analysis as well as DABEST comparisons with respective effect sizes.

	Log (Sum of Coherence)		Sensitivity	1-Specificity	Cut-Off	AUC (95.0%CI)	SE	z	*p*-Value	Hedge’s (95.0%CI)	*p*-Value
	Control	Sarcopenic									
Cz-TA	−6.3 (1.7)	−5.3 (1.5)	0.71	0.75	−5.4 (0.46)	0.68 (0.38–0.98)	0.15	1.17	0.242	0.57 (−0.53–1.52)	0.272
Cz-GL	−6.1 (1.5)	−6.2 (1.4)	1.00	0.14	−7.8/−7.3/−3.5 (0.14) ^b^	0.45 (0.11–0.79)	0.17	−0.29	0.768	−0.02 (−1.20–1.04)	0.798
Cz-GM	−6.6 (1.2)	−5.5 (1.7)	0.82	0.67	−6.1 (0.48)	0.70 (0.45 – 0.95)	0.13	1.54	0.123	0.66 (−0.28–1.61)	0.149
Cz-SOL	−5.5 (1.2)	−4.9 (2.1)	0.63	0.71	−3.8 (0.44)	0.57 (0.24–0.90)	0.17	0.43	0.669	0.35 (−0.75–1.42)	0.685
Cz-VL	−5.6 (1.3)	−5.1 (2.2)	0.44	1.00	−4.9 (0.50)	0.61 (0.31–0.91)	0.15	0.72	0.473	0.2 (−0.7–1.3)	0.517
Cz-VM	−6.9 (1.2)	−3.7 (1.6)	1.00	0.89	−5.2 (0.89)	0.98 (0.92–1.04) ^a^	0.03	15.20	<0.001	2.2 (1.3–3.1)	0.005
Cz-BF	−7.5 (2.1)	−4.5 (1.7)	1.00	0.70	−6.2 (0.70)	0.86 (0.68–1.03) ^a^	0.09	3.97	<0.001	1.5 (0.7–2.2)	0.010
Cz-RF	−7.2 (1.4)	−5.7 (3.0)	0.50	1.00	−4.4 (0.34)	0.70 (0.40–0.99)	0.15	1.32	0.187	0.6 (−0.6–1.6)	0.224

TA = Tibialis Anterior, GL = Gastrocnemius Lateralis, GM = Gastrocnemius Medialis, SOL = Soleus, VL = Vastus Lateralis, VM = Vastus Medialis, BF = Biceps Femoris, RF = Rectus Femoris. ^a^ upper confidence limit can exceed the value of 1.0 in some case, due to the nonparametric estimation method used here of DeLong (1988). ^b^ easyROC calculation of optimal cut-off points yielded to some multiple cut-off points in some cases. In our analysis this was the case only for this parameter, which was, however, not significant.

## References

[1] Clark B.C., Manini T.M. (2008). Sarcopenia≠ dynapenia. J. Gerontol. Ser. A Biol. Sci. Med. Sci..

[2] Falcon L.J., Harris-Love M.O. (2017). Sarcopenia and the New ICD-10-CM Code: Screening, Staging, and Diagnosis Considerations. Fed. Pract..

[3] Rosenberg I.H. (1997). Sarcopenia: Origins and clinical relevance. J. Nutr..

[4] Cruz-Jentoft A.J., Baeyens J.P., Bauer J.M., Boirie Y., Cederholm T., Landi F., Martin F.C., Michel J.P., Rolland Y., Schneider S.M. (2010). Sarcopenia: European consensus on definition and diagnosis: Report of the European Working Group on Sarcopenia in Older People. Age Ageing..

[5] Cruz-Jentoft A.J., Bahat G., Bauer J., Boirie Y., Bruyere O., Cederholm T., Cooper C., Landi F., Rolland Y., Sayer A.A. (2019). Sarcopenia: Revised European consensus on definition and diagnosis. Age Ageing.

[6] Fielding R.A., Vellas B., Evans W.J., Bhasin S., Morley J.E., Newman A.B., Abellan van Kan G., Andrieu S., Bauer J., Breuille D. (2011). Sarcopenia: An undiagnosed condition in older adults. Current consensus definition: Prevalence, etiology, and consequences. International working group on sarcopenia. J. Am. Med. Dir. Assoc..

[7] Morley J.E., Abbatecola A.M., Argiles J.M., Baracos V., Bauer J., Bhasin S., Cederholm T., Coats A.J., Cummings S.R., Evans W.J. (2011). Sarcopenia with limited mobility: An international consensus. J. Am. Med. Dir. Assoc..

[8] Studenski S.A., Peters K.W., Alley D.E., Cawthon P.M., McLean R.R., Harris T.B., Ferrucci L., Guralnik J.M., Fragala M.S., Kenny A.M. (2014). The FNIH sarcopenia project: Rationale, study description, conference recommendations, and final estimates. J. Gerontol. A Biol. Sci. Med. Sci..

[9] Yoshida D., Suzuki T., Shimada H., Park H., Makizako H., Doi T., Anan Y., Tsutsumimoto K., Uemura K., Ito T. (2014). Using two different algorithms to determine the prevalence of sarcopenia. Geriatr. Gerontol. Int..

[10] Bischoff-Ferrari H.A., Orav J.E., Kanis J.A., Rizzoli R., Schlogl M., Staehelin H.B., Willett W.C., Dawson-Hughes B. (2015). Comparative performance of current definitions of sarcopenia against the prospective incidence of falls among community-dwelling seniors age 65 and older. Osteoporos. Int..

[11] Locquet M., Beaudart C., Reginster J.Y., Petermans J., Bruyere O. (2018). Comparison of the performance of five screening methods for sarcopenia. Clin. Epidemiol..

[12] Manini T.M., Visser M., Won-Park S., Patel K.V., Strotmeyer E.S., Chen H., Goodpaster B., De Rekeneire N., Newman A.B., Simonsick E.M. (2007). Knee extension strength cutpoints for maintaining mobility. J. Am. Geriatr. Soc..

[13] von Haehling S., Morley J.E., Anker S.D. (2010). An overview of sarcopenia: Facts and numbers on prevalence and clinical impact. J. Cachexia Sarcopenia Muscle.

[14] Clark B.C., Manini T.M. (2012). What is dynapenia?. Nutrition.

[15] Clark B.C., Manini T.M. (2010). Functional consequences of sarcopenia and dynapenia in the elderly. Curr. Opin. Clin. Nutr. Metab. Care.

[16] Manini T.M., Clark B.C. (2012). Dynapenia and aging: An update. J. Gerontol. A Biol. Sci. Med. Sci..

[17] Kwon Y.N., Yoon S.S. (2017). Sarcopenia: Neurological Point of View. J. Bone Metab..

[18] Manini T.M., Hong S.L., Clark B.C. (2013). Aging and muscle: A neuron’s perspective. Curr. Opin. Clin. Nutr. Metab. Care.

[19] Halliday D.M., Conway B.A., Farmer S.F., Rosenberg J.R. (1998). Using electroencephalography to study functional coupling between cortical activity and electromyograms during voluntary contractions in humans. Neurosci. Lett..

[20] Farmer S.F., Bremner F.D., Halliday D.M., Rosenberg J.R., Stephens J.A. (1993). The frequency content of common synaptic inputs to motoneurones studied during voluntary isometric contraction in man. J. Physiol..

[21] Pfurtscheller G. (1981). Central beta rhythm during sensorimotor activities in man. Electroencephalogr. Clin. Neurophysiol..

[22] Schoffelen J.-M., Oostenveld R., Fries P. (2005). Neuronal coherence as a mechanism of effective corticospinal interaction. Science.

[23] Schoffelen J.M., Poort J., Oostenveld R., Fries P. (2011). Selective movement preparation is subserved by selective increases in corticomuscular gamma-band coherence. J. Neurosci..

[24] Kamp D., Krause V., Butz M., Schnitzler A., Pollok B. (2013). Changes of cortico-muscular coherence: An early marker of healthy aging?. Age.

[25] Johnson A.N., Shinohara M. (2012). Corticomuscular coherence with and without additional task in the elderly. J. Appl. Physiol..

[26] Bayram M.B., Siemionow V., Yue G.H. (2015). Weakening of Corticomuscular Signal Coupling During Voluntary Motor Action in Aging. J. Gerontol. A Biol. Sci. Med. Sci..

[27] Al-Yahya E., Dawes H., Smith L., Dennis A., Howells K., Cockburn J. (2011). Cognitive motor interference while walking: A systematic review and meta-analysis. Neurosci. Biobehav. Rev..

[28] Petersen T.H., Willerslev-Olsen M., Conway B.A., Nielsen J.B. (2012). The motor cortex drives the muscles during walking in human subjects. J. Physiol..

[29] Artoni F., Fanciullacci C., Bertolucci F., Panarese A., Makeig S., Micera S., Chisari C. (2017). Unidirectional brain to muscle connectivity reveals motor cortex control of leg muscles during stereotyped walking. Neuroimage.

[30] Tolea M.I., Galvin J.E. (2015). Sarcopenia and impairment in cognitive and physical performance. Clin. Interv. Aging.

[31] Nishiguchi S., Yamada M., Fukutani N., Adachi D., Tashiro Y., Hotta T., Morino S., Shirooka H., Nozaki Y., Hirata H. (2015). Differential association of frailty with cognitive decline and sarcopenia in community-dwelling older adults. J. Am. Med. Dir. Assoc..

[32] Huang C.Y., Hwang A.C., Liu L.K., Lee W.J., Chen L.Y., Peng L.N., Lin M.H., Chen L.K. (2016). Association of Dynapenia, Sarcopenia, and Cognitive Impairment Among Community-Dwelling Older Taiwanese. Rejuvenation Res..

[33] Hsu Y.H., Liang C.K., Chou M.Y., Liao M.C., Lin Y.T., Chen L.K., Lo Y.K. (2014). Association of cognitive impairment, depressive symptoms and sarcopenia among healthy older men in the veterans retirement community in southern Taiwan: A cross-sectional study. Geriatr. Gerontol. Int..

[34] Ojagbemi A., D’Este C., Verdes E., Chatterji S., Gureje O. (2015). Gait speed and cognitive decline over 2 years in the Ibadan study of aging. Gait Posture.

[35] Gennaro F., de Bruin E.D. (2018). Assessing Brain-Muscle Connectivity in Human Locomotion through Mobile Brain/Body Imaging: Opportunities, Pitfalls, and Future Directions. Front Public Health.

[36] Makeig S., Gramann K., Jung T.-P., Sejnowski T.J., Poizner H. (2009). Linking brain, mind and behavior. Int. J. Psychophysiol..

[37] Bulea T.C., Kim J., Damiano D.L., Stanley C.J., Park H.-S. (2015). Prefrontal, posterior parietal and sensorimotor network activity underlying speed control during walking. Front. Hum. Neurosci..

[38] De Sanctis P., Butler J.S., Malcolm B.R., Foxe J.J. (2014). Recalibration of inhibitory control systems during walking-related dual-task interference: A mobile brain-body imaging (MOBI) study. Neuroimage.

[39] Castermans T., Duvinage M. (2013). Corticomuscular coherence revealed during treadmill walking: Further evidence of supraspinal control in human locomotion. J. Physiol..

[40] Malcolm B.R., Foxe J.J., Butler J.S., De Sanctis P. (2015). The aging brain shows less flexible reallocation of cognitive resources during dual-task walking: A mobile brain/body imaging (MoBI) study. Neuroimage.

[41] Barthelemy D., Willerslev-Olsen M., Lundell H., Conway B.A., Knudsen H., Biering-Sorensen F., Nielsen J.B. (2010). Impaired transmission in the corticospinal tract and gait disability in spinal cord injured persons. J. Neurophysiol..

[42] Larsen L.H., Zibrandtsen I.C., Wienecke T., Kjaer T.W., Christensen M.S., Nielsen J.B., Langberg H. (2017). Corticomuscular coherence in the acute and subacute phase after stroke. Clin. Neurophysiol..

[43] Willerslev-Olsen M., Petersen T.H., Farmer S.F., Nielsen J.B. (2015). Gait training facilitates central drive to ankle dorsiflexors in children with cerebral palsy. Brain.

[44] Roeder L., Boonstra T.W., Kerr G.K. (2020). Corticomuscular control of walking in older people and people with Parkinson’s disease. Sci. Rep..

[45] von Carlowitz-Ghori K., Bayraktaroglu Z., Hohlefeld F.U., Losch F., Curio G., Nikulin V.V. (2014). Corticomuscular coherence in acute and chronic stroke. Clin. Neurophysiol..

[46] Fisher K.M., Zaaimi B., Williams T.L., Baker S.N., Baker M.R. (2012). Beta-band intermuscular coherence: A novel biomarker of upper motor neuron dysfunction in motor neuron disease. Brain.

[47] Jensen P., Frisk R., Spedden M.E., Geertsen S.S., Bouyer L.J., Halliday D.M., Nielsen J.B. (2019). Using Corticomuscular and Intermuscular Coherence to Assess Cortical Contribution to Ankle Plantar Flexor Activity During Gait. J. Mot. Behav..

[48] Spedden M.E., Choi J.T., Nielsen J.B., Geertsen S.S. (2019). Corticospinal control of normal and visually guided gait in healthy older and younger adults. Neurobiol. Aging.

[49] Roeder L., Boonstra T.W., Smith S.S., Kerr G.K. (2018). Dynamics of corticospinal motor control during overground and treadmill walking in humans. J. Neurophysiol..

[50] Spedden M.E., Nielsen J.B., Geertsen S.S. (2018). Oscillatory Corticospinal Activity during Static Contraction of Ankle Muscles Is Reduced in Healthy Old versus Young Adults. Neural. Plast..

[51] Krauth R., Schwertner J., Vogt S., Lindquist S., Sailer M., Sickert A., Lamprecht J., Perdikis S., Corbet T., Millan J.D.R. (2019). Cortico-Muscular Coherence Is Reduced Acutely Post-stroke and Increases Bilaterally During Motor Recovery: A Pilot Study. Front. Neurol..

[52] Doldersum E., van Zijl J.C., Beudel M., Eggink H., Brandsma R., Pina-Fuentes D., van Egmond M.E., Oterdoom D.L.M., van Dijk J.M.C., Elting J.W.J. (2019). Intermuscular coherence as biomarker for pallidal deep brain stimulation efficacy in dystonia. Clin. Neurophysiol..

[53] Michel J.P. (2014). Sarcopenia: There is a need for some steps forward. J. Am. Med. Dir. Assoc..

[54] Gonzalez M.C., Heymsfield S.B. (2017). Bioelectrical impedance analysis for diagnosing sarcopenia and cachexia: What are we really estimating?. J. Cachexia Sarcopenia Muscle.

[55] He Q., Jiang J., Xie L., Zhang L., Yang M. (2018). A sarcopenia index based on serum creatinine and cystatin C cannot accurately detect either low muscle mass or sarcopenia in urban community-dwelling older people. Sci. Rep..

[56] Hars M., Biver E., Chevalley T., Herrmann F., Rizzoli R., Ferrari S., Trombetti A. (2016). Low Lean Mass Predicts Incident Fractures Independently From FRAX: A Prospective Cohort Study of Recent Retirees. J. Bone Miner. Res..

[57] Baumgartner R.N., Koehler K.M., Gallagher D., Romero L., Heymsfield S.B., Ross R.R., Garry P.J., Lindeman R.D. (1998). Epidemiology of sarcopenia among the elderly in New Mexico. Am. J. Epidemiol..

[58] Delmonico M.J., Harris T.B., Lee J.S., Visser M., Nevitt M., Kritchevsky S.B., Tylavsky F.A., Newman A.B., Health A., Body Composition S. (2007). Alternative definitions of sarcopenia, lower extremity performance, and functional impairment with aging in older men and women. J. Am. Geriatr. Soc..

[59] Delmonico M.J., Harris T.B., Visser M., Park S.W., Conroy M.B., Velasquez-Mieyer P., Boudreau R., Manini T.M., Nevitt M., Newman A.B. (2009). Longitudinal study of muscle strength, quality, and adipose tissue infiltration. Am. J. Clin. Nutr..

[60] Chatrian G.E., Lettich E., Nelson P.L. (2015). Ten Percent Electrode System for Topographic Studies of Spontaneous and Evoked EEG Activities. Am. J. EEG Technol..

[61] Hermens H.J., Freriks B., Disselhorst-Klug C., Rau G. (2000). Development of recommendations for SEMG sensors and sensor placement procedures. . J. Electromyogr. Kinesiol..

[62] Oostenveld R., Fries P., Maris E., Schoffelen J.M. (2011). FieldTrip: Open source software for advanced analysis of MEG, EEG, and invasive electrophysiological data. Comput. Intell. Neurosci..

[63] Boonstra T.W., Breakspear M. (2012). Neural mechanisms of intermuscular coherence: Implications for the rectification of surface electromyography. J. Neurophysiol..

[64] Blum S., Jacobsen N.S.J., Bleichner M.G., Debener S. (2019). A Riemannian Modification of Artifact Subspace Reconstruction for EEG Artifact Handling. Front Hum. Neurosci..

[65] Chang C.Y., Hsu S.H., Pion-Tonachini L., Jung T.P. (2019). Evaluation of Artifact Subspace Reconstruction for Automatic Artifact Components Removal in Multi-channel EEG Recordings. IEEE Trans. Biomed. Eng..

[66] Pion-Tonachini L., Hsu S.H., Chang C.Y., Jung T.P., Makeig S. Online automatic artifact rejection using the real-time EEG source-mapping toolbox (REST). Proceedings of the 2018 40th Annual International Conference of the IEEE Engineering in Medicine and Biology Society (EMBC).

[67] Arad E., Bartsch R.P., Kantelhardt J.W., Plotnik M. (2018). Performance-based approach for movement artifact removal from electroencephalographic data recorded during locomotion. PLoS ONE.

[68] Peterson S.M., Ferris D.P. (2019). Group-level cortical and muscular connectivity during perturbations to walking and standing balance. Neuroimage.

[69] Kline J.E., Huang H.J., Snyder K.L., Ferris D.P. (2015). Isolating gait-related movement artifacts in electroencephalography during human walking. J. Neural. Eng..

[70] Nathan K., Contreras-Vidal J.L. (2015). Negligible Motion Artifacts in Scalp Electroencephalography (EEG) During Treadmill Walking. Front. Hum. Neurosci..

[71] Delorme A., Palmer J., Onton J., Oostenveld R., Makeig S. (2012). Independent EEG sources are dipolar. PLoS ONE.

[72] Pion-Tonachini L., Kreutz-Delgado K., Makeig S. (2019). ICLabel: An automated electroencephalographic independent component classifier, dataset, and website. Neuroimage.

[73] Bridwell D.A., Rachakonda S., Silva R.F., Pearlson G.D., Calhoun V.D. (2018). Spatiospectral Decomposition of Multi-subject EEG: Evaluating Blind Source Separation Algorithms on Real and Realistic Simulated Data. Brain Topogr..

[74] Ritterband-Rosenbaum A., Herskind A., Li X., Willerslev-Olsen M., Olsen M.D., Farmer S.F., Nielsen J.B. (2017). A critical period of corticomuscular and EMG-EMG coherence detection in healthy infants aged 9–25 weeks. J. Physiol..

[75] Yoshida T., Masani K., Zabjek K., Chen R., Popovic M.R. (2017). Dynamic cortical participation during bilateral, cyclical ankle movements: Effects of aging. Sci. Rep..

[76] Gennaro F., de Bruin E.D. (2020). A pilot study assessing reliability and age-related differences in corticomuscular and intramuscular coherence in ankle dorsiflexors during walking. Physiol. Rep..

[77] Jensen P., Jensen N.J., Terkildsen C.U., Choi J.T., Nielsen J.B., Geertsen S.S. (2018). Increased central common drive to ankle plantar flexor and dorsiflexor muscles during visually guided gait. Physiol. Rep..

[78] Gwin J.T., Ferris D.P. (2012). Beta- and gamma-range human lower limb corticomuscular coherence. Front. Hum. Neurosci..

[79] Norton J.A., Gorassini M.A. (2006). Changes in cortically related intermuscular coherence accompanying improvements in locomotor skills in incomplete spinal cord injury. J. Neurophysiol..

[80] de Bruin E.D., Patt N., Ringli L., Gennaro F. (2019). Playing Exergames Facilitates Central Drive to the Ankle Dorsiflexors During Gait in Older Adults; a Quasi-Experimental Investigation. Front. Aging Neurosci..

[81] Kitatani R., Ohata K., Aga Y., Mashima Y., Hashiguchi Y., Wakida M., Maeda A., Yamada S. (2016). Descending neural drives to ankle muscles during gait and their relationships with clinical functions in patients after stroke. Clin. Neurophysiol..

[82] Goksuluk D., Korkmaz S., Zararsiz G., Karaagaoglu A.E. (2016). easyROC: An Interactive Web-tool for ROC Curve Analysis Using R Language Environment. R. J..

[83] Ho J., Tumkaya T., Aryal S., Choi H., Claridge-Chang A. (2019). Moving beyond P values: Data analysis with estimation graphics. Nat. Methods..

[84] de Bruin E.D., Schmidt A. (2010). Walking behaviour of healthy elderly: Attention should be paid. Behav. Brain Funct..

[85] Kristeva R., Patino L., Omlor W. (2007). Beta-range cortical motor spectral power and corticomuscular coherence as a mechanism for effective corticospinal interaction during steady-state motor output. Neuroimage.

[86] Mendez-Balbuena I., Huethe F., Schulte-Monting J., Leonhart R., Manjarrez E., Kristeva R. (2012). Corticomuscular coherence reflects interindividual differences in the state of the corticomuscular network during low-level static and dynamic forces. Cereb. Cortex.

[87] Mima T., Toma K., Koshy B., Hallett M. (2001). Coherence between cortical and muscular activities after subcortical stroke. Stroke.

[88] Fang Y., Daly J.J., Sun J., Hvorat K., Fredrickson E., Pundik S., Sahgal V., Yue G.H. (2009). Functional corticomuscular connection during reaching is weakened following stroke. Clin. Neurophysiol..

[89] Rossiter H.E., Eaves C., Davis E., Boudrias M.H., Park C.H., Farmer S., Barnes G., Litvak V., Ward N.S. (2012). Changes in the location of cortico-muscular coherence following stroke. Neuroimage Clin..

[90] Zheng Y., Peng Y., Xu G., Li L., Wang J. (2017). Using Corticomuscular Coherence to Reflect Function Recovery of Paretic Upper Limb after Stroke: A Case Study. Front. Neurol..

[91] Belardinelli P., Laer L., Ortiz E., Braun C., Gharabaghi A. (2017). Plasticity of premotor cortico-muscular coherence in severely impaired stroke patients with hand paralysis. Neuroimage Clin..

[92] Caviness J.N., Adler C.H., Sabbagh M.N., Connor D.J., Hernandez J.L., Lagerlund T.D. (2003). Abnormal corticomuscular coherence is associated with the small amplitude cortical myoclonus in Parkinson’s disease. Mov. Disord..

[93] Clark B.C., Woods A.J., Clark L.A., Criss C.R., Shadmehr R., Grooms D.R. (2019). The Aging Brain & the Dorsal Basal Ganglia: Implications for Age-Related Limitations of Mobility. Adv. Geriatr. Med. Res..

[94] Wu T., Hallett M., Chan P. (2015). Motor automaticity in Parkinson’s disease. Neurobiol. Dis..

[95] Casati M., Costa A.S., Capitanio D., Ponzoni L., Ferri E., Agostini S., Lori E. (2019). The Biological Foundations of Sarcopenia: Established and Promising Markers. Front. Med..

[96] Witham C.L., Riddle C.N., Baker M.R., Baker S.N. (2011). Contributions of descending and ascending pathways to corticomuscular coherence in humans. J. Physiol..

[97] Bijlsma J.W., Berenbaum F., Lafeber F.P. (2011). Osteoarthritis: An update with relevance for clinical practice. Lancet.

[98] Felson D.T., Naimark A., Anderson J., Kazis L., Castelli W., Meenan R.F. (1987). The prevalence of knee osteoarthritis in the elderly. The Framingham Osteoarthritis Study. Arthritis Rheum..

[99] Guccione A.A., Felson D.T., Anderson J.J., Anthony J.M., Zhang Y., Wilson P.W., Kelly-Hayes M., Wolf P.A., Kreger B.E., Kannel W.B. (1994). The effects of specific medical conditions on the functional limitations of elders in the Framingham Study. Am. J. Public Health.

[100] Centers for Disease Control and Prevention (2009). Prevalence and most common causes of disability among adults--United States, 2005. MMWR Morb. Mortal. Wkly. Rep..

[101] Woolf A.D., Pfleger B. (2003). Burden of major musculoskeletal conditions. Bull World Health Organ..

[102] Berger M.J., Chess D.G., Doherty T.J. (2011). Vastus medialis motor unit properties in knee osteoarthritis. BMC Musculoskelet. Disord..

[103] Fink B., Egl M., Singer J., Fuerst M., Bubenheim M., Neuen-Jacob E. (2007). Morphologic changes in the vastus medialis muscle in patients with osteoarthritis of the knee. Arthritis Rheum..

[104] Tieland M., Trouwborst I., Clark B.C. (2018). Skeletal muscle performance and ageing. J. Cachexia Sarcopenia Muscle.

[105] Gandevia S.C. (2001). Spinal and supraspinal factors in human muscle fatigue. Physiol. Rev..

[106] Taylor J.L. (2009). Point: The interpolated twitch does/does not provide a valid measure of the voluntary activation of muscle. J. Appl. Physiol..

[107] Clark B.C., Taylor J.L. (2011). Age-related changes in motor cortical properties and voluntary activation of skeletal muscle. Curr. Aging Sci..

[108] Harridge S.D.R., Kryger A., Stensgaard A. (1999). Knee extensor strength, activation, and size in very elderly people following strength training. Muscle Nerve.

[109] Stevens J.E., Stackhouse S.K., Binder-Macleod S.A., Snyder-Mackler L. (2003). Are voluntary muscle activation deficits in older adults meaningful?. Muscle Nerve.

[110] Clark D.J., Fielding R.A. (2012). Neuromuscular contributions to age-related weakness. J. Gerontol. A. Biol. Sci. Med. Sci..

[111] Fields D.P., Roberts B.M., Simon A.K., Judge A.R., Fuller D.D., Mitchell G.S. (2019). Cancer cachexia impairs neural respiratory drive in hypoxia but not hypercapnia. J. Cachexia Sarcopenia Muscle.

[112] Clark B.C. (2019). Neuromuscular Changes with Aging and Sarcopenia. J Frailty Aging.

[113] Tsekoura M., Billis E., Tsepis E., Dimitriadis Z., Matzaroglou C., Tyllianakis M., Panagiotopoulos E., Gliatis J. (2018). The Effects of Group and Home-Based Exercise Programs in Elderly with Sarcopenia: A Randomized Controlled Trial. J. Clin. Med..

[114] Yoshimura Y., Wakabayashi H., Yamada M., Kim H., Harada A., Arai H. (2017). Interventions for Treating Sarcopenia: A Systematic Review and Meta-Analysis of Randomized Controlled Studies. J. Am. Med. Dir. Assoc..

[115] Unhjem R., Lundestad R., Fimland M.S., Mosti M.P., Wang E. (2015). Strength training-induced responses in older adults: Attenuation of descending neural drive with age. Age.

[116] Unhjem R., Nygard M., van den Hoven L.T., Sidhu S.K., Hoff J., Wang E. (2016). Lifelong strength training mitigates the age-related decline in efferent drive. J. Appl. Physiol..

[117] Franzon K., Zethelius B., Cederholm T., Kilander L. (2019). The impact of muscle function, muscle mass and sarcopenia on independent ageing in very old Swedish men. BMC Geriatr.

[118] Eggenberger P., Wolf M., Schumann M., de Bruin E.D. (2016). Exergame and balance training modulate prefrontal brain activity during walking and enhance executive function in older adults. Front. Aging Neurosci..

[119] McCaskey M.A., Schattin A., Martin-Niedecken A.L., de Bruin E.D. (2018). Making More of IT: Enabling Intensive Motor Cognitive Rehabilitation Exercises in Geriatrics Using Information Technology Solutions. Biomed. Res. Int..

[120] Schättin A., Arner R., Gennaro F., de Bruin E.D. (2016). Adaptations of prefrontal brain activity, executive functions, and gait in healthy elderly following exergame and balance training: A randomized-controlled study. Front. Aging Neurosci..

[121] Nagano Y., Ishida K., Tani T., Kawasaki M., Ikeuchi M. (2016). Short and long-term effects of exergaming for the elderly. Springerplus.

[122] Sato K., Kuroki K., Saiki S., Nagatomi R. (2015). Improving Walking, Muscle Strength, and Balance in the Elderly with an Exergame Using Kinect: A Randomized Controlled Trial. Games Health J..

[123] Peterson S.J., Braunschweig C.A. (2016). Prevalence of Sarcopenia and Associated Outcomes in the Clinical Setting. Nutr. Clin. Pract..

